# From Screening to a Nanotechnological Platform: Cannabidiol–Chemotherapy Co-Loaded Lipid Nanocapsules for Glioblastoma Multiforme Treatment

**DOI:** 10.3390/pharmaceutics17121537

**Published:** 2025-11-29

**Authors:** Laura Gómez-Lázaro, Juan Aparicio-Blanco, Ana Isabel Fraguas-Sánchez, María Consuelo Montejo-Rubio, Cristina Martín-Sabroso, Ana Isabel Torres-Suárez

**Affiliations:** 1 Department of Pharmaceutics and Food Technology, Faculty of Pharmacy, Complutense University of Madrid, Pl Ramón y Cajal s/n, 28040 Madrid, Spain; lgomez14@ucm.es (L.G.-L.); juan.aparicio.blanco@ucm.es (J.A.-B.); aifraguas@ucm.es (A.I.F.-S.); 2 Institute of Industrial Pharmacy, Faculty of Pharmacy, Complutense University of Madrid, Pl Ramón y Cajal s/n, 28040 Madrid, Spain; 3 Department of Health and Pharmaceutical Sciences, School of Pharmacy, Universidad San Pablo-CEU, CEU Universities, Urbanización Montepríncipe, 28660 Boadilla del Monte, Spain; montejo@ceu.es

**Keywords:** glioma, cannabinoids, chemotherapy, co-delivery, nanomedicine

## Abstract

**Background/Objective**: Cannabidiol (CBD) has gained increasing interest due to its multifaceted anticancer properties and favourable safety profile. Glioblastoma multiforme (GBM), a highly aggressive brain tumour with limited treatment options, represents a compelling target for CBD-based therapies. In this study, we report the rational design of two distinct formulations of lipid nanocapsules (LNCs) co-encapsulating CBD and a chemotherapeutic agent, tailored for intracranial and systemic administration. **Methods**: The cytotoxicity of various CBD–chemotherapeutic combinations, including temozolomide, carmustine, doxorubicin, and paclitaxel (PTX), were screened in vitro in U-87 MG and U-373 MG human GBM cell lines and analyzed for chemical compatibility. Moreover, the efficacy and the anti-migratory effect of the selected combination was further assessed in ovo and in vitro, respectively. Lastly, two LNC formulations coloaded with the selected combination were prepared in two different sizes via the phase inversion temperature method. **Results**: First, CBD in solution exhibited potent cytotoxicity and significantly inhibited cell migration in both GBM cell lines. Among the CBD–chemotherapeutic combinations tested, only CBD + PTX demonstrated both additive/synergistic interaction and favourable chemical compatibility. Second, this enhanced effect was confirmed in ovo. Third, the CBD + PTX combination also exhibited anti-migratory effect. Finally, two co-loaded LNC formulations—51.2 ± 0.9 nm and 25.9 ± 0.3 nm in size—were developed for intracranial and systemic delivery, respectively. Both formulations exhibited high monodispersity, a slightly negative ζ-potential, and consistently maintained a 7.5:1 CBD:PTX mass encapsulation ratio across both particle sizes. **Conclusions**: CBD + PTX co-loaded LNCs represent a promising and versatile nanomedicine platform for GBM therapy.

## 1. Introduction

In recent years, cannabinoids have gained attention in cancer pharmacotherapy not only for their utility in palliative care but also for their potential effects on cancer progression and their favourable safety profiles compared with traditional cytostatic drugs [1,2]. Emerging evidence suggests that cannabinoids may exert multifactorial antitumour effects, including induction of apoptosis, inhibition of angiogenesis, and impairment of tumour cell migration across diverse cancer types, such as prostate, breast, liver, colon, pancreatic or myeloma [3,4,5,6,7,8]. Glioblastoma multiforme (GBM) also stands out as a compelling target for cannabinoid-based therapeutic strategies given its high proliferative index, extensive angiogenesis, and aggressive clinical behaviour [9,10]. GBM is one of the most lethal primary brain tumours, characterized by dismal prognosis (median survival of 14.6 months), frequent recurrence, and a therapeutic landscape largely limited to surgical resection followed by radio-chemotherapy with temozolomide (TMZ) [11,12,13].

The therapeutic potential of cannabinoids in glioma has been progressively substantiated by a growing body of clinical evidence. Early studies focused on Δ^9^-tetrahydrocannabinol (THC), the main cannabinoid present in Cannabis sativa. A pilot clinical trial in the early 2000s demonstrated that the intratumoural THC administration (Phase I, completed) in recurrent GBM patients was safe and showed preliminary antiproliferative effects [14]. In the following studies, the standardized extract of nabiximols, composed of THC and cannabidiol (CBD) in a 1.08:1 mass ratio, was used. CBD, the most abundant non-psychoactive phytocannabinoid, is credited with mitigating the psychoactive effects of THC [15]. Thus, throughout the 2010s, clinical research focused on evaluating the tolerability, safety and adverse event profile of nabiximols combined with the chemotherapeutic agent TMZ in patients with recurrent GBM (NCT01812603, Phase Ib, completed; NCT01812616, Phase Ib, completed) [16]. The promising results obtained led to the design of studies evaluating the efficacy of these combination therapies using the THC:CBD mixture. Two active clinical trials are currently investigating this approach. The GEINOCANN trial (NCT03529448, Phase Ib/II, active) is assessing the safety and efficacy of a 1:1 CBD: THC solution administered alongside radiotherapy and TMZ in newly diagnosed GBM patients [17]. Similarly, the ARISTOCRAT trial (NCT05629702, Phase II, recruiting) is exploring whether nabiximols with standard TMZ treatment can improve overall survival in patients with recurrent, MGMT promoter-methylated GBM, a molecular subtype associated with TMZ sensitivity [18]. Interest in CBD has increased in recent years due to its antitumour effects observed in preclinical studies and its lack of psychoactive properties, which facilitates its availability for both research and clinical use. Two clinical trials are currently underway to evaluate the efficacy of a CBD solution in sesame oil for prostate cancer (NCT04428203, Phase I) and breast cancer (NCT05016349, Phase III).

It should be noted that in all these clinical trials, the CBD or THC + CBD formulations were administered sublingually, buccally or orally. However, the bioavailability of these cannabinoids through these routes of administration is very low and variable. Indeed, the oral bioavailability of CBD is approximately 6–10%, mainly due to its limited aqueous solubility, erratic intestinal absorption and extensive hepatic first-pass metabolism [19,20], while oromucosal bioavailability is slightly higher, ranging from 13% to 35%, but still highly variable [21]. This could explain the inconclusive results observed in the clinical trials already completed, despite promising preclinical outcomes, where cannabinoids are typically administered intraperitoneally or subcutaneously. Moreover, due to its limited aqueous solubility, CBD requires the use of organic solvents in these formulations, which increases their toxicity and restrains their applicability for parenteral administration.

This highlights the need for rational formulation strategies guided by biopharmaceutical criteria to reduce the likelihood of subsequent clinical failures. In this context, nanotechnology-based delivery systems have emerged as promising platforms to avoid the use of organic solvents and promote the accumulation of CBD at the tumour site. This is particularly advantageous in GBM, where chemoresistance and the restrictive blood–brain barrier (BBB) severely impair therapeutic efficacy.

Notably, nanomedicine also enables the co-delivery of multiple drugs. Unlike current CBD, THC and TMZ combinations being tested in clinical trials, wherein distribution relies on the intrinsic pharmacokinetic properties of each drug, since each one is typically administered via distinct routes and dosage forms, co-encapsulation within a single nanocarrier can synchronize their distribution. This strategic alignment may ultimately enhance synergistic effects at the tumour site. Multi-drug nanomedicine has already been reported to facilitate the co-delivery of anticancer agents [22]. In addition to synchronizing the pharmacokinetics of encapsulated drugs, ensuring that both drugs reach the tumour simultaneously, this approach can also reduce systemic toxicity by limiting off-target exposure, which is particularly relevant in glioblastoma therapy, where systemic toxicity limits the dose of antineoplastic agent that can be administered.

Building on this rationale, nanomedicine offers an unprecedented opportunity to co-encapsulate CBD along with chemotherapeutic agents to not only maximize their synergistic therapeutic effect but also minimize the development of resistance mechanisms. Specifically, lipid nanocapsules (LNCs) are promising carriers for this purpose for several reasons. First, they are formulated using excipients classified as Generally Recognized as Safe (GRAS) by regulatory authorities, reinforcing their potential for clinical translation. These excipients can be customized to meet the solubility requirements of distinct drug substances. Second, the LNC preparation procedure is a solvent-free method that enables precise control over particle size, allowing rational customization for various routes of administration [23]. Third, LNCs support the efficient co-delivery of both hydrophilic and lipophilic compounds [24]. Based on previous findings by Aparicio-Blanco et al. [25], which demonstrated the efficient encapsulation of CBD in LNCs, the present study proposes the development of a co-loaded LNC system incorporating CBD and a chemotherapeutic agent selected through prior comprehensive biological and chemical screening.

**Figure 1:**
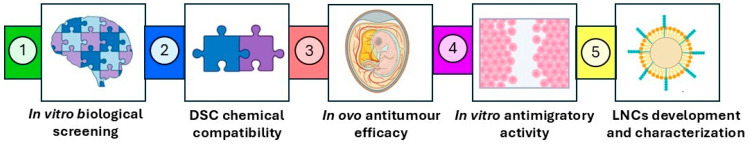
Schematic diagram summarizing the workflow of the study.

Hence, in this study, we present a rational design for a CBD-based combination therapy in GBM, structured in two sequential steps: (1) biological and chemical screening to identify the most synergistic and compatible CBD-chemotherapy combination for co-delivery; and (2) the formulation and characterization of two co-loaded LNC-based systems, aimed at enabling tailored therapeutic interventions for GBM via intracranial or systemic administration. This stepwise design (Figure 1), which, to the best of our knowledge, has not been previously reported, is intended to optimize delivery efficiency and therapeutic performance, ultimately contributing to the development of safer and more effective cannabinoid-based strategies for GBM treatment, while enhancing their translational potential.

## 2. Materials and Methods

### 2.1. Cell Lines and Culture Conditions

The human glioblastoma cell lines U-87 MG and U-373 MG were purchased from the American Type Culture Collection cell bank (ATCC^®^ HTB-14™, Manassas, VA, USA) and from the European Collection of Authenticated Cell Culture (ECACC^®^ 08061901, Salisbury, UK), respectively. Cells were cultured in Dulbecco’s Modified Eagle Medium (DMEM, Gibco, Life Technologies, Waltham, MA, USA) supplemented with 10% foetal bovine serum (FBS; Gibco, Life Technologies) and 1% penicillin (10,000 U/mL) and streptomycin (10,000 μg/mL) solution (Gibco, Life Technologies). Cells were cultured as adherent monolayers in 75 cm^2^ flasks (Corning^®^, Corning, NY, USA) at 37 °C with 5% CO_2_ in a humidified atmosphere. The experiments were initiated with cells at passage 12, and all assays were completed within the following five passages.

### 2.2. In Vitro Cytotoxicity Assay in U-87 MG and U-373 MG Cells

Cell viability of U-87 MG and U-373 MG cells was assessed in the presence of five different drugs: cannabidiol (CBD; THC Pharma, Frankfurt am Main, Germany), paclitaxel (PTX; Sigma Aldrich, St. Louis, MO, USA), doxorubicin hydrochloride (DOX; Sigma Aldrich), carmustine (BCNU, Sigma-Aldrich) and temozolomide (TMZ; TCl, Tokyo, Japan).

#### 2.2.1. In Vitro Monotherapy Studies

The cytotoxic effects of free CBD, PTX, DOX, BCNU and TMZ on cell proliferation were evaluated in the human glioblastoma cell lines U-87 MG and U-373 MG. Cells were seeded in 96-well plates at a density of 10^4^ cells/well. After 24 h, the culture medium was removed and replaced with treatments at the following concentration ranges: CBD (5 × 10^−1^–10^2^ µM), TMZ (10^2^–10^4^ µM), BCNU (1–10^3^ µM), DOX (10^−3^–10^3^ µM) and PTX (5 × 10^−6^–5 × 10^3^ µM). At 24 and 48 h post-treatment, the medium was discarded and replaced with 200 µL of a 3-(4,5-dimethyl-2-thiazolyl)-2,5-diphenyl-2H-tetrazolium bromide (MTT; Sigma Aldrich) solution (0.5 mg/mL) in complete DMEM medium. Cells were incubated with the MTT solution for 3 h. Subsequently, the MTT solution was removed, and 200 µL of DMSO was added to each well to dissolve the resulting formazan crystals. Plates were shaken for 10 min, and absorbance was measured at 570 nm using a Varioskan Lux microplate reader (Thermo Scientific, Waltham, MA, USA). Complete DMEM medium was used as a negative control. The IC_50_ values were calculated for each treatment. Experiments were performed in quadruplicate with four replicates per condition in each one (N = 4, n = 4).

#### 2.2.2. In Vitro Combination Studies

The combination of CBD at concentrations of 15 and 25 µM with varying concentrations of TMZ (10^2^–5 × 10^3^ µM), BCNU (1–7.5 × 10^2^ µM), DOX (10^−3^–10^3^ µM) and PTX (5 × 10^−6^–5 × 10^3^ µM) was also investigated. U-87 MG and U-373 MG glioma cells were seeded in 96-well plates at a density of 10^4^ cells/well and treated 24 h after seeding with the respective drug combinations (CBD + PTX, CBD + DOX, CBD + BCNU and CBD + TMZ). Cell viability was assessed at 24 and 48 h post-treatment using the MTT method as described in the section on in vitro monotherapy studies. The assay was carried out in four different experiments with four replicates per condition in each one (N = 4, n = 4).

To evaluate the potential synergistic effects of the different drug combinations, the SynergyFinder online tool (https://synergyfinder.fimm.fi, accessed on 15 July 2025) was used to compare cell viability data from combination therapies with those from the corresponding monotherapies [26]. Both 2D and 3D synergy maps were generated to illustrate the therapeutic potential of each combination. Additionally, the Bliss synergy score was calculated under the assumption of independent mechanism of action of both drugs in each combination [27]. A synergy score between −10 and +10 suggests an additive effect, scores greater than +10 suggest synergy and scores less than −10 indicate antagonism. Bliss scores above 0 are represented in red on the heat maps, whereas Bliss scores below 0 are depicted in green [28].

### 2.3. Chemical Compatibility Between CBD and Chemotherapeutics

Differential scanning calorimetry (DSC) analyses were performed to evaluate the potential chemical compatibility between CBD and each of the chemotherapeutic drugs at a 1:1 mass ratio. DSC scans were carried out using a Mettler TA 4000 DSC 3 Star System (Mettler Toledo, Columbus, OH, USA) calibrated with indium. Samples were weighed (~3 mg) directly into perforated aluminium pans and heated at a rate of 10 °C/min from −20 to 300 °C under a nitrogen flow of 20 mL/min.

### 2.4. Wound Healing Assay

A scratch-wound healing assay was conducted to investigate the effect of CBD, PTX and CBD: PTX on glioblastoma cell migration. U-87 MG (2 × 10^5^ cells/well) and U-373 MG (1.8 × 10^5^ cells/well) were seeded in 12-well plates (Corning^®^). Cells were incubated for 24 h until a homogeneous monolayer was formed (~90% confluence). To inhibit cell proliferation, the complete DMEM was replaced with DMEM supplemented with mitomycin C (25 µg/mL; Fisher Scientific, Waltham, MA, USA), a blocker of DNA synthesis, and incubated for 30 min [29]. Afterwards, a linear scratch was made in each well using a 200 µL pipette tip. The medium was then removed, and wells were rinsed with 300 µL of PBS to remove detached cells. Then, free CBD (5, 10, 15 µM), PTX (5 × 10^−3^, 10^−2^, 1.5 × 10^−2^ µM) and CBD: PTX (5:5 × 10^−3^; 10:10^−2^ and 15:1.5 × 10^−2^ µM) were added. Non-cytotoxic concentrations were selected in all cases to ensure that any changes observed in migratory behaviour could be attributed to the drugs themselves, rather than being secondary to cell death. The assay was performed in triplicate with two analytical replicates (N = 3; n = 2).

Images of the wound area were captured at 0, 6, and 24 h post-treatment using a Leica DMIL LED Fluo microscope to monitor gap closure. Wound areas were analysed using the ImageJ 1.53t software, and the percentage of wound closure at each time point was calculated using the following equation:

(1) $$Wound\;closure\;\left(\%\right)=\frac{A_{0}-A_{t}}{A_{0}}\times 100$$

where A_0_ is the initial wound area at t = 0 and A_t_ is the wound area at either t = 6 or 24 h post-treatment.

### 2.5. Antitumour Efficacy Using the Hen Egg Test-Chorioallantoic Membrane (HET-CAM) Assay

The chorioallantoic membrane (CAM) model, utilizing fertilized white Leghorn hen eggs (HET-CAM), was used to assess the antitumour efficacy of CBD, PTX, and their combination in solution on U-87 MG and U-373 MG derived tumours. Briefly, the fertilized eggs were incubated in vertical position in a rotatory automated incubator at 37 °C and 50% relative humidity (Avimac^®^ Serie C-160). On the tenth embryonic day of development (EDD 10), the CAM was gently detached from the eggshell, and a 3-mm window was opened. Afterwards, the CAM was gently scratched and U-87 MG or U-373 MG cells (2 × 10^6^ or 10^6^ cells, respectively) suspended in 50 µL of Geltrex™ Reduced Growth Factor Basement Membrane Matrix (Gibco) were deposited within a 12-mm silicone O-ring placed on the CAM to restrict lateral diffusion. By EDD 13, visible U-87 MG/U-373 MG-derived tumours had formed. The initial tumour area (TA) was determined by capturing images and analysing them using Image J software. Afterwards, eggs were divided into four treatment groups and tumours were topically treated with: (i) complete DMEM medium, which served as a control, (ii) CBD in solution (100 µM), (iii) PTX in solution (0.1 µM) and (iv) a combination of CBD and PTX (100:0.1 µM), with at least six eggs per condition (N ≥ 6). Following treatment, eggs were returned to the incubator until EDD 15. At EDD 15, final tumour area images were captured, and tumours were excised for weight measurement. The tumour growth was calculated using the following equation:

(2) $$Tumor\;growth\;\left(\%\right)=\frac{{TA}_{f}}{{TA}_{i}}\times 100$$

where TA_i_ is the area measured at EDD13 (pre-treatment) and TA_f_ is the area measured at EDD15 (48 h-post treatment). No specific authorization was required for in ovo experiments in accordance with European and Spanish legislation (Directive 2010/63/EU and Royal Decree 53/2013, respectively).

### 2.6. Preparation of LNCs

#### 2.6.1. Blank-LNCs

**Figure 2:**
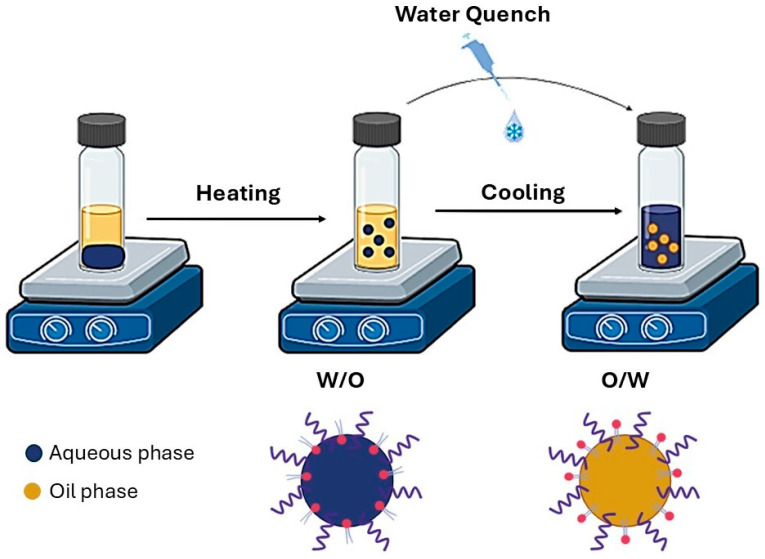
Scheme of the phase inversion temperature (PIT) method.

Blank-LNCs were prepared by the low-energy phase inversion temperature (PIT) method [25]. In this process, a mixture of polyethylene glycol (15)–hydroxystearate (Kolliphor^®^ HS15; Sigma-Aldrich), soybean phospholipids with 70% phosphatidylcholine (Lipoid^®^ S75, Lipoïd, Ludwigshafen, Germany), medium-chain triglycerides of caprylic and capric acids (Labrafac^®^ lipophile WL1349; Gattefossé, Saint-Priest, France), NaCl (Panreac, Barcelona, Spain) and MiliQ water (Millipore, Burlington, MA, USA) was prepared under magnetic stirring. The mixture was then progressively heated to 90 °C, exceeding the PIT of the system, to ensure lipid melting. Subsequently, the mixture was gradually cooled down until the PIT was reached. A rapid quenching step with cold water (5 mL) was then applied, resulting in the formation of the final LNC suspension (Figure 2). Blank-LNCs were prepared in two different sizes by adjusting the oil-to-surfactant (i.e., Kolliphor^®^ HS15-Labrafac^®^ lipophile WL1349) mass ratio.

#### 2.6.2. CBD and PTX Co-Loaded LNCs

To formulate the CBD and PTX co-loaded LNCs (CBD.PTX-LNCs), both drugs were first dissolved in the Labrafac lipophile WL1349, which forms the oily core of the LNCs, by adding 200 µL of acetone. Following this, the acetone was evaporated, and the remaining excipients were added. The mixture was then gradually heated and cooled around the PIT as described in Section 2.6.1. The co-loaded LNCs were prepared in two distinct sizes by varying the oil-to-surfactant mass ratio. In all cases, the theoretical drug-to-oil mass ratio was 15 and 2% (*w*/*w*) for CBD and PTX, respectively.

### 2.7. Characterization of LNCs

#### 2.7.1. Size Distribution

The particle size and polydispersity index (PDI) of Blank-LNCs and CBD.PTX-LNCs were determined using dynamic light scattering (DLS) with a Zetasizer Blue Lab (Malvern Instruments, Malvern, UK). The measurements were conducted at 25 °C on LNC dispersions, which were diluted to a concentration of 3.90 mg of excipients/mL in ultrapure water to ensure optimal scattering conditions. Three independent batches of each formulation were analysed.

#### 2.7.2. ζ-Potential

The ζ-potential of both Blank and CBD.PTX-LNCs was measured by electrophoretic light scattering (ELS) using the same instrument as for size distribution. The measurements were conducted at 25 °C on LNC dispersions that were diluted to a concentration of 0.39 mg of excipients/mL in saline solution (0.58 mg of NaCl/mL). All measurements were performed on three different batches of each formulation.

#### 2.7.3. Morphology

The morphology of the co-loaded LNCs (F2 and F4) was examined by transmission electron microscopy (TEM) using a JEM 1400 microscope operated at 120 kV. For TEM samples preparation, 20 µL of an aqueous LNC suspension (2 mg/mL) was placed into a 200-mesh carbon-coated copper grid. The samples were negatively stained with 2% (*w*/*v*) uranyl acetate for F2 and 1% (*w*/*v*) phosphotungstic acid for F4 and dried at room temperature before imaging.

#### 2.7.4. Encapsulation Efficiency and Drug Content

The encapsulation efficiency (EE) of CBD and PTX within the LNCs was indirectly calculated by subtracting the amount of unencapsulated drug, determined through centrifugal ultrafiltration using 100 kDa Amicon^®^ Centrifugal filters (6440 g, 60 min), from the total amount of CBD and PTX present in the suspension. The total drug content was obtained after breaking the LNCs with methanol (1:60 *v*/*v*). This process was repeated across three independent batches of each formulation.

CBD and PTX co-loading in CBD.PTX-LNCs were quantified by high-performance liquid chromatography with UV detection (HPLC–UV). The HPLC-UV method was adapted from Aparicio-Blanco et al. [25] and Jaradat et al. [30] using an Agilent 1200 Infinity equipment. A mixture of acetonitrile: water (60:40) at a flow rate of 1.3 mL/min was used as the mobile phase under isocratic conditions. Separation was achieved using a reversed-phase Col Kromaphase 100^®^ C18 (5 μm, 15 × 0.46 cm, Scharlau) column. Detection was carried out at a wavelength of 223 nm for both analytes, and the injection volume was set at 20 µL. The EE was calculated using Equation 3, while the drug loading (DL) was calculated using Equation (4):

(3) $$EE\left(\%\right)=\frac{Total\;amount\;of\;drug-amount\;of\;unencapsulated\;drug}{Total\;amount\;of\;drug}\times 100$$

(4) $$DL\left(\%\right)=\frac{Total\;amount\;of\;drug-amount\;of\;unencapsulated\;drug}{Weight\;of\;nanocapsules}\times 100$$

### 2.8. Statistical Analysis

All experiments were done at least in triplicate, and all data are expressed as mean ± standard error of the mean (SEM). N corresponds to the number of independent experiments performed, while n is the total number of replicates for each experiment. Two-way ANOVA followed by a post hoc Tukey’s multiple comparison test was used for the migration assay. Two-way ANOVA followed by a post hoc Dunnett’s multiple comparison test was applied to the in vitro combination studies. IC_50_ values were calculated through non-linear regression with a sigmoidal (four-parameter logistic) model. One-way ANOVA followed by a post hoc Tukey’s multiple comparison test was used for the in ovo and migration studies. Two-way ANOVA was used for the characterization of the LNCs in terms of PDI and ζ-potential, and a t-test for the statistical evaluation of size, EE and DL. In all cases, statistical significance was fixed as *: *p* <0.05, **: *p* < 0.01, ***: *p* < 0.001, ***: *p* < 0.0001. All analyses were conducted using the GraphPad Prism 10.4.2 software.

## 3. Results and Discussion

### 3.1. Evaluation of CBD as a Modulator of Proliferation and Migration in GBM

Due to the absence of psychoactive effects, CBD remains a promising therapeutic compound. It has demonstrated multiple pharmacological properties in various cancer types, including prostate, breast, lung, colon, pancreatic and myeloma [7,31,32,33,34,35], such as antiproliferative, pro-apoptotic, anti-migratory, and anti-invasive effects, as well as modulation of the tumour microenvironment [36]. In this study, the antitumour activity of CBD has been evaluated in two human glioma cell lines: U-87 MG, characterised by high proliferative and invasive capacity, and U-373 MG, which exhibits a less invasive phenotype. A comparative approach has been used to determine whether CBD exerts consistent antitumour effects across GBM models with distinct biological behaviours.

**Figure 3:**
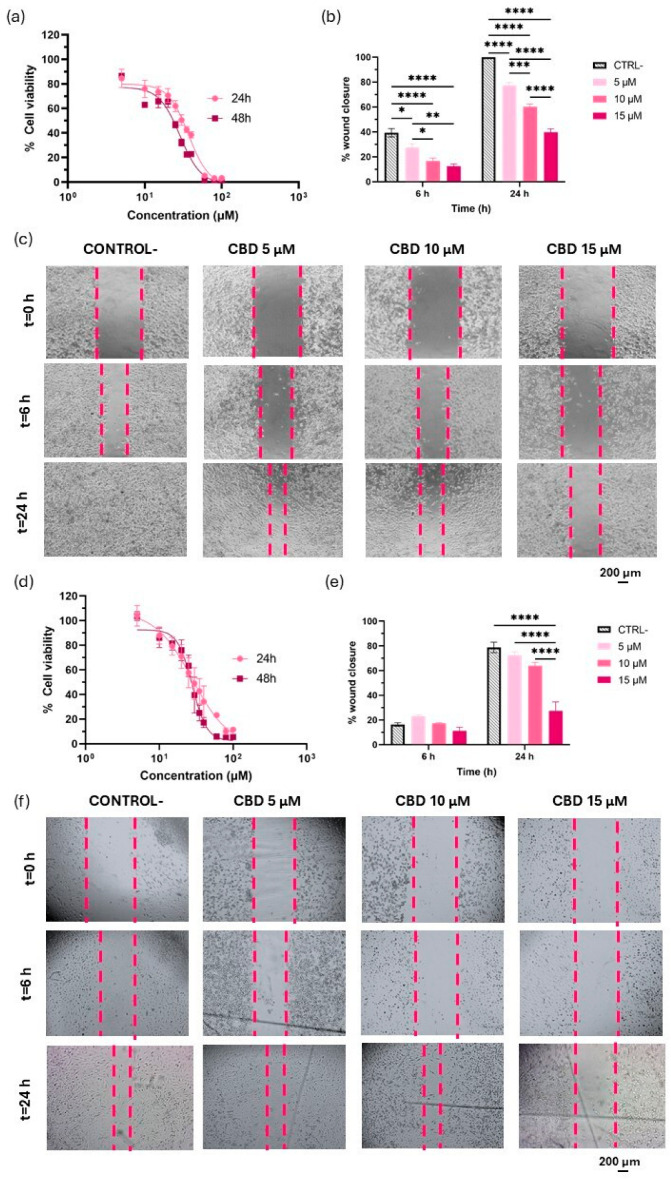
Effect of CBD on cell viability and migration in U-87 MG and U-373 MG glioma cell lines. (**a**) Cytotoxicity of CBD in solution against the U-87 MG cell line; (**b**) Effect of CBD at 5, 10, and 15 µM on U-87 MG cell migration at 6 and 24 h, assessed by wound closure percentage; (**c**) Representative images of scratched U-87 MG cell monolayers for each treatment condition and time point; (**d**) Cytotoxicity of CBD in solution against the U-373 MG cell line; (**e**) Effect of CBD at 5, 10, and 15 µM on U-373 MG cell migration at 6 and 24 h, assessed by wound closure percentage; (**f**) Representative images of scratched U-373 MG cell monolayers for each treatment condition and time point. Scalebar: 200 µm. Statistical analysis: two-way ANOVA followed by a post hoc Tukey’s multiple comparison test. Statistical significance is shown only for the treatment factor: * *p* < 0.05; ** *p* < 0.01; *** *p* < 0.001; **** *p* < 0.0001.

CBD inhibits the proliferation of both U-87 MG and U-373 MG human glioma cell lines in a concentration-dependent manner (Figure 3a,d). Notably, in U-87 MG cells, CBD also exhibited time-dependent cytotoxicity, with IC_50_ values of 37 µM at 24 h and 28 µM after 48 h of incubation (Table 1). These values are consistent with those previously reported by Nabissi et al. [37]. In contrast, CBD did not show a time-dependent antiproliferative effect in U-373 MG cells with IC_50_ values of 28 µM at 24 h and 27 µM at 48 h, closely aligning with the findings of Massi et al. [38] and Aparicio-Blanco et al. [25]. Importantly, no statistically significant differences in IC_50_ values were observed between the two cell lines at either timepoint. This suggests that while the antiproliferative effect of CBD may increase over time in more aggressive glioma phenotypes like U-87 MG, there are no statistically significant differences in the overall efficacy after prolonged exposure between the two cell models. These data support the in vitro antitumour efficacy of CBD against human glioblastoma. These results are consistent with studies investigating the effects of CBD on other human glioblastoma cell lines, such as U251 [39], T98 [40] and SF126 [41]. Additionally, the antitumour potential of CBD has also been explored in murine glioma cell lines, including C6 [32] and GL261 [42].

Building upon the evidence that CBD has antiproliferative activity, its potential to impair GBM cell migration was evaluated, as infiltration into surrounding brain tissue represents one of the most aggressive and treatment-resistant features of GBM [43,44]. For this purpose, the wound healing assay was used, namely a simple, rapid and cost-effective method commonly used to study cell migration [45,46].

Cell migration into the scratched area was monitored microscopically over 24 h (Figure 3c,f). In the U-87 MG cell line, CBD exhibited an anti-migratory effect at all tested concentrations (5, 10 and 15 µM) as early as 6 h post-treatment, compared to the control group. This early response is likely due to the inherently higher migratory capacity of these cells, which facilitates the early detection of CBD-induced effects on cell migration. The inhibitory effect on migration persisted for 24 h and followed an evident concentration-dependent pattern (Figure 3b,c). Whereas control cells achieved complete wound closure (100%) within 24 h, wound closure only reached 77.31 ± 4.0%, 60.16 ± 1.04% and 39.75 ± 4.54% for 5, 10 and 15 µM of CBD, respectively, over the same period.

Conversely, in U-373 MG cells, control cells achieved 16.36 ± 2.47% wound closure at 6 h, thereby preventing detection of any CBD-induced effects on migration at this early time point. Remarkably, even after 24 h, control U-373 MG cells did not reach complete wound closure (78.81 ± 4.73%), in alignment with their less invasive phenotype. However, while 5 and 10 µM CBD still did not produce a significant antimigratory effect at this time, 15 µM CBD significantly inhibited cell migration, with only 23.46 ± 12.44% wound closure (Figure 3e,f).

Overall, these findings indicate that CBD effectively impairs GBM cell migration in both cell lines, with a greater effect observed in the highly invasive U-87 MG cells. The earlier effect observed with U-87 MG cells could be attributed to the higher intrinsic invasive capacity of this cell line over U373-MG. These results underscore the potential of CBD as a multi-target therapeutic agent in GBM, capable of inhibiting both tumour proliferation and migration.

### 3.2. Biological and Chemical Screening of CBD and Chemotherapy Combinations

The effectiveness of chemotherapy in GBM remains severely limited by the frequent development of resistance. Several factors contribute to this challenge, including the highly infiltrative nature of GBM, as well as its pronounced genetic and cellular heterogeneity [47]. Given the ability of CBD to reduce both cell proliferation and migration, its potential in combination with standard chemotherapeutics was investigated next. The aim was to assess whether co-administration could enhance antitumour efficacy while enabling dose reduction of conventional drugs, thereby minimizing toxicity and improving therapeutic outcomes. While in vitro cytotoxicity is commonly emphasized during early preformulation stages, the evaluation of drug–drug compatibility is often overlooked. To address this, DSC analysis was performed to evaluate the chemical compatibility between CBD and each of the selected chemotherapeutic agents. This compatibility assessment is a critical step in the context of co-delivery systems, where two or more drugs are encapsulated together within the same nanocarrier to ensure the same biodistribution upon administration. Unlike other combination therapies in which drugs may be administered separately and interact only transiently in the biological milieu, co-loaded formulations require that both compounds remain chemically stable in proximity, not only during administration and distribution, but also throughout manufacturing and storage. Ensuring such compatibility is essential to maintain the integrity and therapeutic performance of the formulation over its entire shelf-life and represents a foundational aspect of translational research.

TMZ, an alkylating agent approved by the FDA and EMA in 2005 and 2009, respectively, for the treatment of adult patients with newly diagnosed GBM, remains the first-line chemotherapy for GBM combined with surgical resection and radiotherapy [12,13]. Due to ongoing trials combining CBD: THC with TMZ (NCT03529448 and NCT05629702), TMZ was selected as the reference cytotoxic agent to be tested alongside CBD in this study.

At 24 and 48 h, the IC_50_ values of TMZ were comparable between both glioma cell lines (Table 1). However, in the U-87 MG cell line, TMZ exhibited cytotoxic activity in a time-dependent manner (2.1 × 10^3^ μM and 1.3 × 10^3^ μM after 24 and 48 h, respectively), although in all cases IC_50_ values were in the millimolar range.

**Figure 4:**
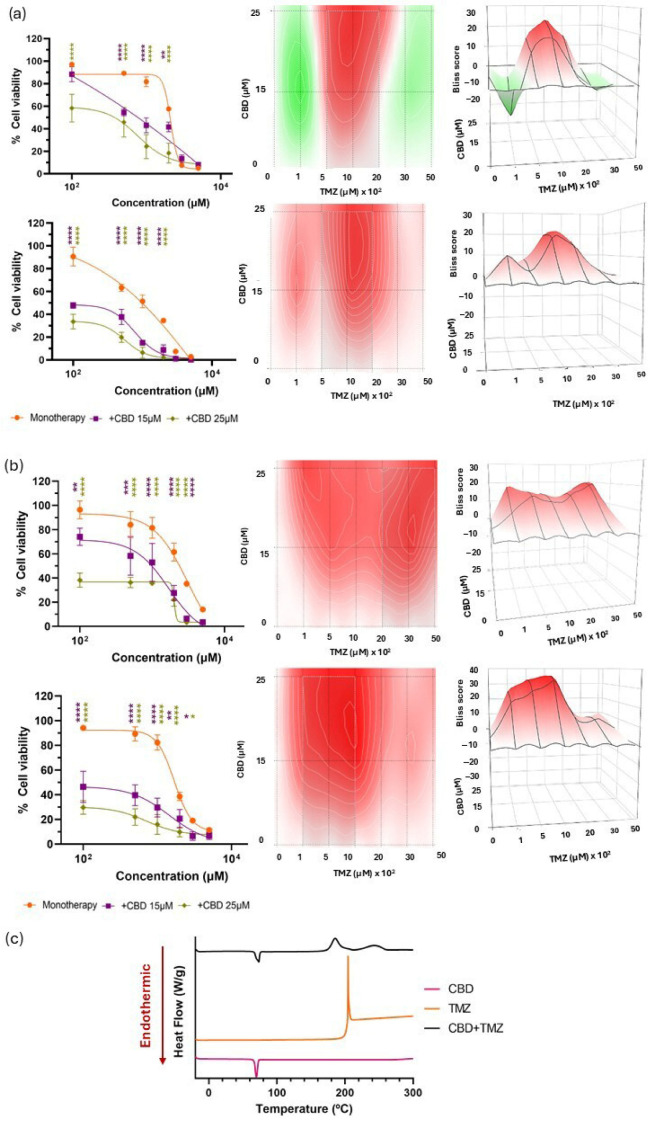
Evaluation of the biological and chemical effects of CBD in combination with TMZ. (**a**) Cell viability following combination therapies with CBD and TMZ compared with that of TMZ in monotherapy at 24 h (upper row, left) and 48 h (lower row, left) in U-87 MG cell line. 2D (centre) and 3D (right) synergy maps generated with SynergyFinder 3.0 using the Bliss model at each time point; (**b**) Cell viability following combination therapies with CBD and TMZ compared with that of TMZ in monotherapy at 24 h (upper row, left) and 48 h (lower row, left) in U-373 MG cell line. 2D (centre) and 3D (right) synergy maps generated with SynergyFinder 3.0 using the Bliss model at each time point. All results were normalized to untreated control cells and expressed as mean ± SEM (N = 4, n = 4). Statistical analysis: two-way ANOVA followed by a post hoc Dunnett’s multiple comparison test. Statistical significance is shown only for the treatment factor using TMZ in monotherapy as the reference group: * *p* < 0.05; ** *p* < 0.01; *** *p* < 0.001; **** *p* < 0.0001. Bliss score legend: <−10: antagonistic effect; (−10, 10): additive effect; >10: synergistic effect. Bliss scores above 0 are represented in red on the heat maps, whereas Bliss scores below 0 are depicted in green; (**c**) DSC thermograms of CBD, TMZ and their 1:1 mass ratio mixture.

In vitro analysis of the CBD and TMZ combination revealed a significantly enhanced reduction in cell viability in both U-87 MG and U-373 MG glioma cell lines, particularly when higher concentrations of CBD (25 µM) were combined with lower concentrations of TMZ (Figure 4a,b). This suggests a potential for dose reduction of TMZ, thereby minimizing associated toxicity. These findings are consistent with previous work by Nabissi et al. [37]. The effect was time-dependent and became more pronounced at 48 h. At 24 h, Bliss synergy scores indicated an additive interaction in U-87 MG cell line with a Bliss score of 3.98 ± 3.72 and a synergistic effect in U-373 MG with a Bliss score of 13.76 ± 4.78. At 48 h, Bliss scores rose to 9.66 ± 2.46 in U-87 MG, indicating a slightly additive synergistic effect, and to 20.20 ± 3.74 in U-373 MG, reflecting an enhanced synergistic effect (Table 2). The observed synergy likely stems from the complementary mechanisms of action of CBD and TMZ, reinforcing the potential of this combination to enhance therapeutic efficacy in GBM treatment while reducing TMZ-related toxicity.

In terms of physicochemical characterization, DSC analysis revealed a single exothermic peak for TMZ at 207.03 °C, consistent with its thermal degradation profile previously reported by Ahad et al. [48]. In the case of CBD, a well-defined endothermic peak was observed at 69.45 °C, corresponding to its melting point, in agreement with prior findings by Li et al. [49] and by Vlad et al. [50]. However, the DSC thermogram of the binary mixture (Figure 4c) revealed a splitting of the characteristic exothermic peak of TMZ into two distinct exothermic events in the presence of CBD. This suggests a possible interaction between the two compounds, potentially leading to the formation of multiple degradation products with different thermal characteristics. These results indicate a lack of physicochemical compatibility between CBD and TMZ under the tested conditions, which may pose challenges for their co-encapsulation within a single delivery system. Therefore, alternative combinations with more favourable compatibility profiles were explored to fully leverage the benefits of co-delivery systems.

Moving forward in the pursuit of effective therapeutic alternatives for GBM, BCNU, a chemotherapeutic agent formerly included in its gold-standard treatment as local therapy [51], was selected as a second candidate based on its DNA-alkylating mechanism, which closely resembles that of TMZ. Therefore, it was hypothesized that a similar synergistic effect might be achieved with BCNU while preserving drug compatibility. Additionally, the clinical use of BCNU is limited by its short plasma half-life and systemic toxicity, which, taken together, make BCNU a suitable candidate for benefiting from encapsulation within nanocarriers.

First, in vitro results demonstrated a more potent antiproliferative effect for BCNU compared to TMZ (Table 1). U-87 MG cells only exhibited greater sensitivity to BCNU than U-373 MG cells after 48 h, with IC_50_ values of 1.7 × 10^2^ µM and 2.7 × 10^2^ µM, respectively. No time-dependency was evidenced in monotherapy with none of the glioma cell lines.

**Figure 5:**
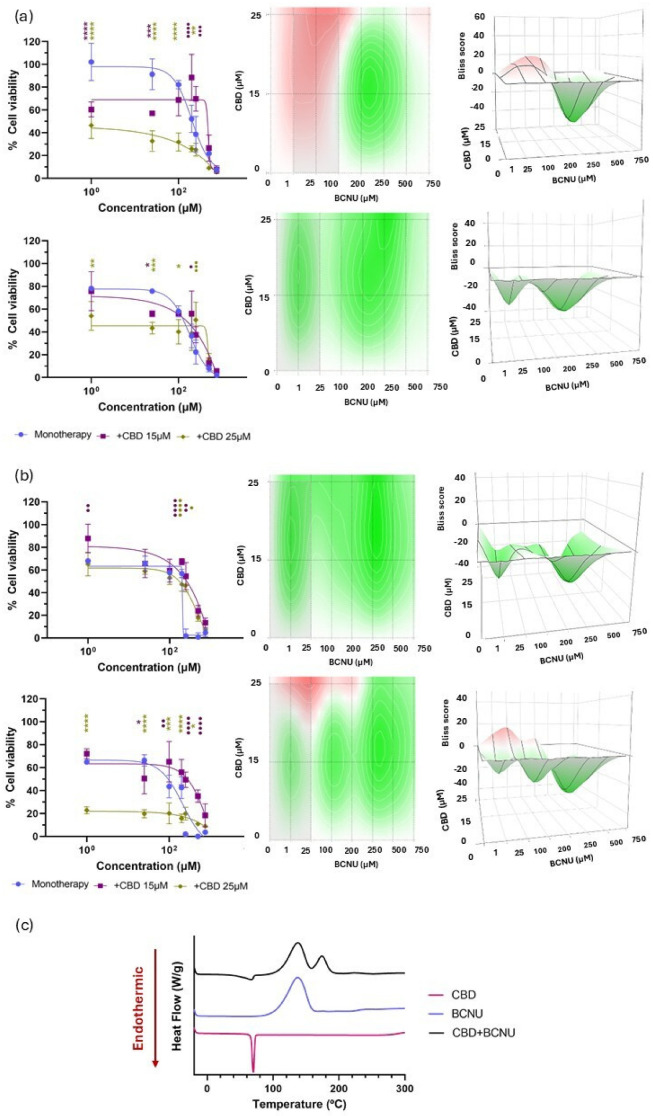
Evaluation of the biological and chemical effects of CBD in combination with BCNU. (**a**) Cell viability following combination therapies with CBD and BCNU compared with that of BCNU in monotherapy at 24 h (upper row, left) and 48 h (lower row, left) in U-87 MG cell line. 2D (centre) and 3D (right) synergy maps generated with SynergyFinder 3.0 using the Bliss model at each time point; (**b**) Cell viability following combination therapies with CBD and BCNU compared with that of BCNU in monotherapy at 24 h (upper row, left) and 48 h (lower row, left) in U-373 MG cell line. 2D (centre) and 3D (right) synergy maps generated with SynergyFinder 3.0 using the Bliss model at each time point. All results were normalized to untreated control cells and expressed as mean ± SEM (N = 4, n = 4). Statistical analysis: two-way ANOVA followed by a post hoc Dunnett’s multiple comparison test. Statistical significance is shown only for the treatment factor using BCNU in monotherapy as the reference group: * *p* < 0.05; ** *p* < 0.01; *** *p* < 0.001; **** *p* < 0.0001 (additive effect). ● *p* < 0.05; ●● *p* < 0.01; ●●● *p* < 0.001; ●●●● *p* < 0.0001 (antagonistic effect). Bliss score legend: <−10: antagonistic effect; (−10, 10): additive effect; >10: synergistic effect. Bliss scores above 0 are represented in red on the heat maps, whereas Bliss scores below 0 are depicted in green; (**c**) DSC thermograms of CBD, BCNU and their 1:1 mass ratio mixture.

When combining CBD with BCNU in U-87 MG cells, two regions could be observed at both timepoints: whereas at lower concentrations of BCNU an additive effect was evidenced, at higher concentrations of BCNU an antagonistic effect was revealed. As a result of this antagonistic effect at higher BCNU concentrations, Bliss scores reached negative values of −2.25 ± 5.42 at 24 h and −19.10 ± 5.07 at 48 h (Figure 5a). In the case of U-373 MG cells, a time-dependent effect on the combination therapy was also displayed, with an antagonistic effect observed after 24 h, whereas at 48 h an additive effect emerged at higher concentrations of CBD. This time-dependency was supported by the Bliss score values that shifted from a strong antagonistic effect at 24 h (Bliss score: −24.37 ± 4.02) to an additive/slightly antagonistic interaction after 48 h (Bliss score: −9.89 ± 4.23) (Figure 5b).

The overall antagonistic interaction was further supported by DSC analysis, which revealed that, apart from the characteristic exothermic peak of BCNU at 137.55 °C [52], an additional exothermic peak emerged when combined with CBD at 174.67 °C. This thermal behaviour suggests that BCNU undergoes degradation in the presence of CBD (Figure 5c). Due to the lack of positive effects, this combination was not considered further in this study.

Given the failure of the combinations of CBD with TMZ and BCNU, the two standard chemotherapeutics used in GBM treatment, new combinations were explored with alternative chemotherapeutic agents that have not yet been considered in the clinical setting for GBM. The third alternative tested was DOX. Although the exact mechanism of action of DOX remains controversial, it is known to exert its cytotoxic effects through multiple pathways, including DNA intercalation and adduct formation, inhibition of topoisomerase II, generation of free radicals and oxidative stress, and disruption of cellular membranes [53]. On the one hand, the antitumour efficacy of DOX in GBM is limited by its poor BBB crossing properties, owing to P-glycoprotein (P-gp) efflux [54]. On the other hand, its clinical use is limited by its risk of cardiotoxicity [55]. The rationale for exploring the combination of CBD and DOX in GBM lies not only in their individual antineoplastic properties but also in preclinical evidence indicating that CBD may reduce DOX-induced cardiotoxicity when co-administered intraperitoneally in animal models [56].

As shown in Table 1, DOX exhibited a stronger antiproliferative effect than the previous alkylating agents, with comparable potency in both glioma cell lines. Notably, the antiproliferative effect of DOX followed a clear time-dependent pattern in both cell lines. For U-87 MG, the IC_50_ decreased from 61 µM at 24 h to 3.5 × 10^−1^ µM at 48 h, while for U-373 MG this value was reduced from 6.64 µM at 24 h to 8.9 × 10^−1^ µM at 48 h.

**Figure 6:**
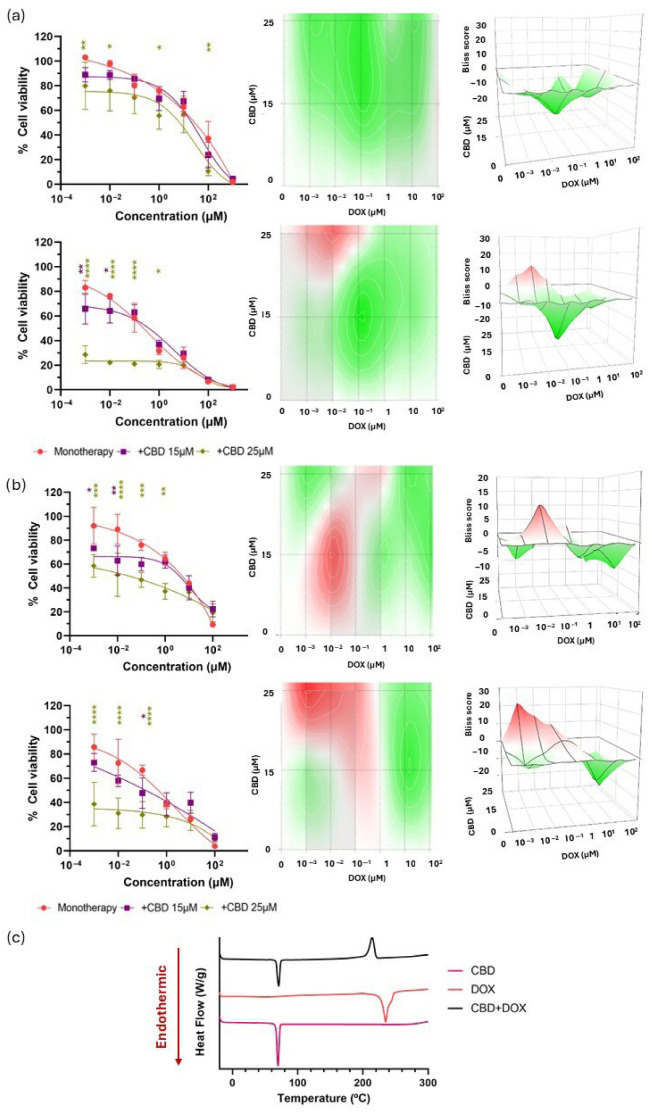
Evaluation of the biological and chemical effects of CBD in combination with DOX. (**a**) Cell viability following combination therapies with CBD and DOX compared with that of DOX in monotherapy at 24 h (upper row, left) and 48 h (lower row, left) in U-87 MG cell line. 2D (centre) and 3D (right) synergy maps generated with SynergyFinder 3.0 using the Bliss model at each time point; (**b**) Cell viability following combination therapies with CBD and DOX compared with that of DOX in monotherapy at 24 h (upper row, left) and 48 h (lower row, left) in U-373 MG cell line. 2D (centre) and 3D (right) synergy maps generated with SynergyFinder 3.0 using the Bliss model at each time point. All results were normalized to untreated control cells and expressed as mean ± SEM (N = 4, n = 4). Statistical analysis: two-way ANOVA followed by a post hoc Dunnett’s multiple comparison test. Statistical significance is shown only for the treatment factor using DOX in monotherapy as the reference group: * *p* < 0.05; ** *p* < 0.01; *** *p* < 0.001; **** *p* < 0.0001. Bliss score legend: <−10: antagonistic effect; (−10, 10): additive effect; >10: synergistic effect. Bliss scores above 0 are represented in red on the heat maps, whereas Bliss scores below 0 are depicted in green; (**c**) DSC thermograms of CBD, DOX and their 1:1 mass ratio mixture.

In agreement with the time-dependency evidenced in DOX monotherapy, this effect was likewise present in combination therapy in both cell lines and particularly for the highest CBD concentrations (Figure 6a,b). This time-dependency was also supported by the Bliss score values in both cell lines (Table 2). The shift in Bliss score values was especially relevant for U-87 MG cells, where Bliss scores shifted from a strong antagonistic effect at 24 h (−17.03 ± 6.03) to an additive interaction after 48 h (−5.12 ± 3.63).

Furthermore, DSC analysis of the CBD + DOX combination revealed a thermal shift indicative of physicochemical incompatibility. The characteristic endothermic melting peak of DOX at 234.43 °C, previously reported by Gao et al. [57] and associated with its crystalline structure, was replaced by an exothermic event. This transition suggests a loss of crystallinity and potential degradation of DOX in the presence of CBD (Figure 6c). Overall, although CBD may offer cardioprotective effects, its combination with DOX did not improve therapeutic efficacy and could even be counterproductive, limiting its potential for future studies.

Finally, PTX, a microtubule-stabilizing agent known to induce mitotic arrest and cell death [58], was evaluated. On the one hand, the antitumour efficacy of PTX in GBM is critically hampered by its inability to reach effective concentrations in the central nervous system, largely due to its high affinity for the P-gp efflux transporter [59,60], which has led to its exclusion from standard GBM treatment protocols. Furthermore, taxanes, particularly PTX, are associated with a high incidence of peripheral neuropathy, affecting approximately 60–70% of patients undergoing chemotherapy [61,62]. However, preclinical studies have shown that cannabinoids confer neuroprotective effects against paclitaxel-induced peripheral neuropathy, preventing mechanical and thermal allodynia, preserving neurite outgrowth in sensory neurons, and reducing neuronal damage via mechanisms involving 5-HT_1A_ and CB_2_ receptors. Therefore, studying the combination of CBD and PTX not only holds potential for enhancing the cytotoxicity of PTX against tumour cells but also for mitigating its neurotoxicity [63,64].

Among all compounds tested in this study, PTX showed the highest potency, reaching the lowest IC_50_ values among all tested drugs (Table 1). In U-87 MG cells, the antiproliferative effect was time-dependent, with the IC_50_ decreasing nearly 250-fold from 15 µM at 24 h to 5.9 × 10^−2^ µM at 48 h. In U-373 MG cells, although PTX also exhibited strong antiproliferative activity, no time-dependent trend was observed.

**Figure 7:**
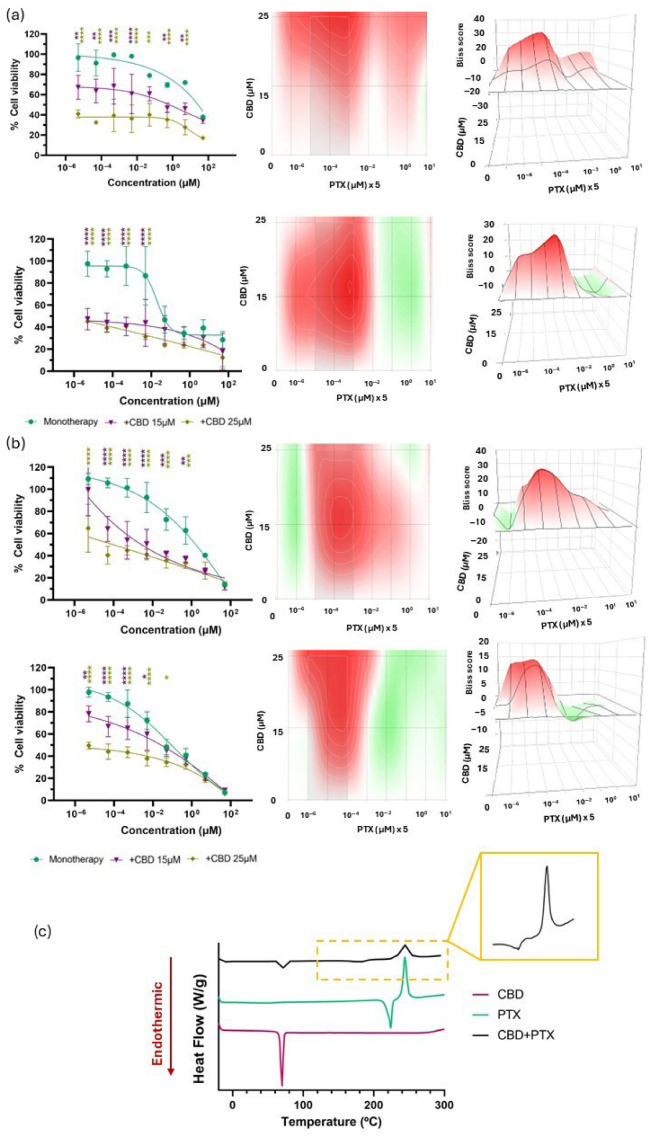
Evaluation of the biological and chemical effects of CBD in combination with PTX. (**a**) Cell viability following combination therapies with CBD and PTX compared with that of PTX in monotherapy at 24 h (upper row, left) and 48 h (lower row, left) in U-87 MG cell line. 2D (centre) and 3D (right) synergy maps generated with SynergyFinder 3.0 using the Bliss model at each time point; (**b**) Cell viability following combination therapies with CBD and PTX compared with that of PTX in monotherapy at 24 h (upper row, left) and 48 h (lower row, left) in U-373 MG cell line. 2D (centre) and 3D (right) synergy maps generated with SynergyFinder 3.0 using the Bliss model at each time point. All results were normalized to untreated control cells and expressed as mean ± SEM (N = 4, n = 4). Statistical analysis: two-way ANOVA followed by a post hoc Dunnett’s multiple comparison test. Statistical significance is shown only for the treatment factor using PTX in monotherapy as the reference group: * *p* < 0.05; ** *p* < 0.01; *** *p* < 0.001; **** *p* < 0.0001. Bliss score legend: <−10: antagonistic effect; (−10, 10): additive effect; >10: synergistic effect. Bliss scores above 0 are represented in red on the heat maps, whereas Bliss scores below 0 are depicted in green; (**c**) DSC thermograms of CBD, PTX and their 1:1 mass ratio mixture.

The combination of CBD and PTX resulted in a synergistic interaction in both glioma cell lines, with enhanced cytotoxic effects particularly evident at higher concentrations of CBD and lower concentrations of PTX (Figure 7a,b). With regard to the Bliss scores, in the U-87 MG cell line, the combination therapy showed a synergistic effect at 24 h (Bliss score: 11.58 ± 5.49) that shifted into an additive effect after 48 h (Bliss score: 6.62 ± 5.49). For the U-373 MG cell line, an additive effect is maintained over the 48 h period. These findings suggest that the combined treatment could allow for a reduction in the effective dose of PTX, potentially minimizing its dose-limiting peripheral neuropathy while preserving antitumour efficacy.

Furthermore, the DSC analysis (Figure 7c) revealed that the characteristic thermal peaks of PTX—an endothermic peak at 223.68 °C, corresponding to its melting point and a secondary exothermic transition around 244.26 °C, corresponding to its degradation temperature [65], remained clearly identifiable in the thermogram of the binary mixture, indicating the absence of significant interactions between the two compounds. Taken together, the additive/synergistic antitumour activity observed in vitro and the confirmed thermal compatibility support the CBD + PTX combination as the most promising candidate. Consequently, subsequent studies focused on this combination to further explore its therapeutic potential in ovo.

### 3.3. In Ovo Evaluation of Antitumour Efficacy in the HET-CAM Model Using U-87 MG and U-373 MG Glioblastoma Xenografts

Given the additive/synergistic antiproliferative effect of the combination of CBD and PTX observed in vitro, along with their compatibility for co-delivery demonstrated by DSC analysis, their antitumour effects were subsequently assessed in the in ovo HET-CAM model. The HET-CAM model has emerged as a compelling alternative to conventional in vivo assays for cancer research, providing a biologically relevant and ethically sound platform for evaluating growth and metastatic potential of tumours, angiogenesis, and the antitumour efficacy of novel therapies, circumventing the need for animal experimentation [66,67]. This model supports tumour induction directly on the chorioallantoic membrane, providing a simple, reproducible, and cost-effective approach. A key advantage of this model lies in the immunological immaturity of the chick embryo, which lacks fully established immune mechanisms during early stages of development. As a result, human tumour cells can be implanted without triggering graft rejection, enabling the formation of xenogeneic tumours. This approach has been successfully applied to generate tumours derived from various cancer types, including lung, pancreatic, melanoma, ovarian and breast cancer [68,69,70,71]. In the case of glioma, while the model has been used to generate tumours, to the best of our knowledge, it has not yet been applied to evaluate the antitumour efficacy of cytotoxic drugs in glioma xenografts, neither as monotherapy nor as combination therapy. For the evaluation of new antitumour treatments, the drugs can be administered intravenously into the main blood vessel of the CAM or, more frequently, topically administered. Although this model has also been proposed as an alternative to rodent studies to evaluate the pharmacokinetics and biodistribution of pharmaceuticals, these studies are generally limited to analysing interactions with the vasculature of the CAM or tumour neovascularization. The absence of a developed hepatic metabolism, mature organs and physiological barriers (such as kidneys, liver, blood–brain barrier, mucous membranes, etc.), and an adaptive immune system, in addition to significant anatomical differences with mammals, are the main factors limiting this application [72].

**Figure 8:**
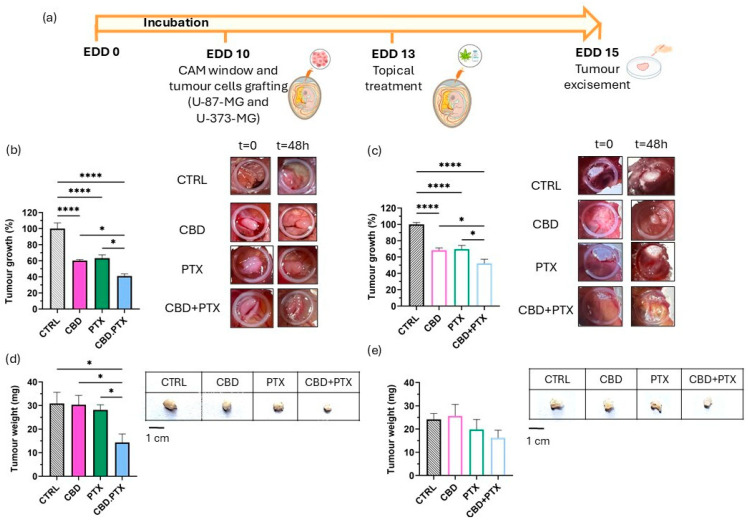
Evaluation of the antitumour effects of CBD, PTX, and their combination (in solution) in the HET-CAM model using U-87 MG and U-373 MG glioma-derived tumours. (**a**) Experimental timeline for the in ovo HET-CAM model using U-87 MG and U-373 MG glioma-derived tumours; (**b**,**c**) Tumour area analysis 48 h post-treatment referred to EDD13 (day of treatment), along with representative images for each treatment group at EDD13 and EDD15 (48 h post-treatment) of the U-87 MG and U-373 MG tumours formed on the CAM membrane, respectively. Results were normalized to untreated control tumours and expressed as mean ± SEM (N ≥ 5); (**d**,**e**) Tumour weight analysis 48 h after treatment, along with representative images for each treatment group of excised U-87 MG and U-373 MG tumours, respectively. Scalebar: 1 cm. Statistical analysis: one-way ANOVA followed by a post hoc Tukey’s multiple comparison test. * *p* < 0.05; **** *p* < 0.0001.

In this study, HET-CAM model has been used to verify the synergistic effect of the CBD + PTX combination therapy following topical administration as an intermediate model between in vitro and in vivo models. A single dose of CBD, PTX, or their combination (CBD + PTX) was administered 72 h after tumour cell implantation, with tumour progression evaluated 48 h post-treatment (Figure 8a).

In U-87 MG-derived tumours, both CBD and PTX as monotherapies led to significant reductions in tumour growth of 39.86 ± 3.67% and 36.71 ± 9.33%, respectively, when compared to untreated controls. Notably, the combination of CBD and PTX resulted in a statistically significant reduction in tumour growth in comparison with monotherapies with each drug alone. The tumour growth reduction was 59.05 ± 6.97%, which nearly doubled the effect of either agent alone (Figure 8b). This enhanced effect was corroborated by tumour weight measurements at EDD15, where the combination group showed the lowest tumour mass (14.34 ± 9.56 mg) versus 30.30 ± 8.88 mg for CBD, 28.15 ± 6.10 mg for PTX and 30.85 ± 11.71 mg for the control group (Figure 8d).

In the U-373 MG-derived tumour model, both CBD and PTX also demonstrated significant antitumour effects when administered as monotherapies, reducing tumour growth by 30.14 ± 7.83% and 30.15 ± 11.86%, respectively. Their combination resulted in a greater tumour growth inhibition of 47.67 ± 12.28%, which was statistically superior to either treatment alone (Figure 8c), as it occurred in U-87 MG-derived in ovo xenografts. However, while a downward trend in tumour weight was observed in the combination group, no statistically significant differences were detected among treatment conditions (Figure 8e), indicating a more moderate therapeutic response in this cell line compared with the U-87 MG cell line.

Taken together, these results underscore the CBD and PTX combination as a highly effective strategy against GBM, demonstrating additive/synergistic antitumour effects in ovo, particularly in the U-87 MG cell line, which aligns with previous results obtained in vitro. These findings not only support its potential as a promising candidate for further development in co-delivery systems but also highlight its significant translational potential for improving therapeutic outcomes in GBM.

### 3.4. PTX and CBD + PTX Anti-Migratory Activity

As already demonstrated in this study, CBD plays a significant role in inhibiting GBM cell migration. Building on this finding and considering the promising synergistic cytotoxic effects of the CBD + PTX combination observed in vitro and in ovo, we further investigated the impact of both PTX and the CBD + PTX combination on GBM cell migration. Evaluating these anti-migratory effects alongside cytotoxicity and in ovo antitumour activity provides a more comprehensive assessment of their therapeutic potential, capturing benefits that cannot be inferred solely from cell viability or tumour growth measurements.

**Figure 9:**
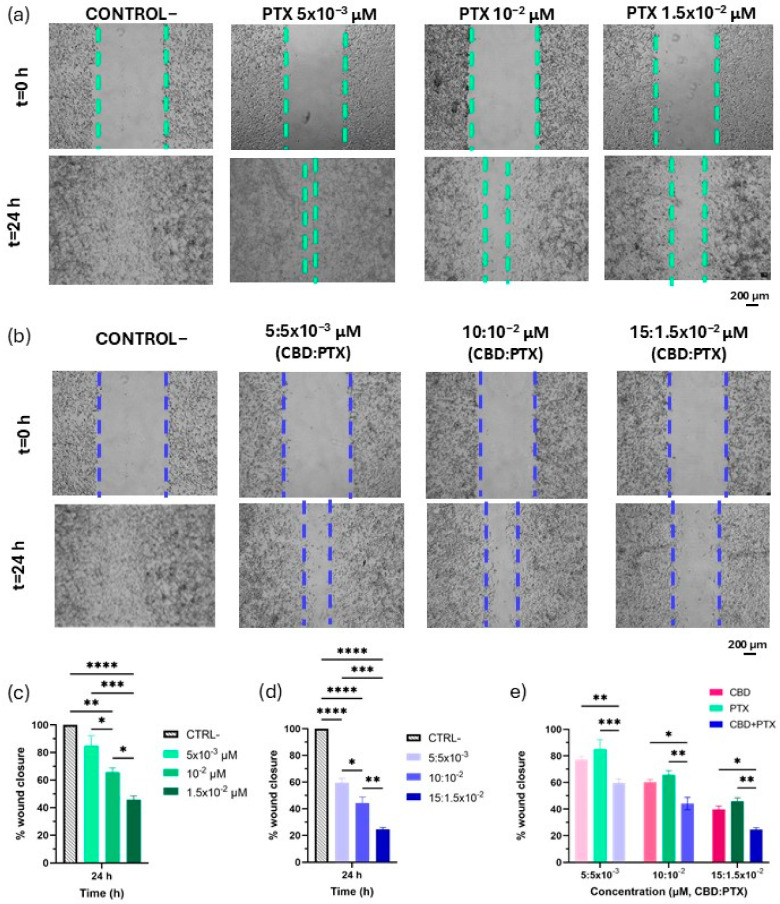
Effect of PTX and CBD + PTX on migration in U-87 MG glioma cell line. (**a**) Representative images of scratched U-87 MG cell monolayers for each PTX concentration (5 × 10^−3^, 10^−2^, 1.5 × 10^−2^ µM) and time point; (**b**) Representative images of scratched U-87 MG cell monolayers for each CBD:PTX concentration (5:5 × 10^−3^, 10:10^−2^, 15:1.5 × 10^−2^ µM) and time point; (**c**) Effect of PTX at 5 × 10^−3^ (light green), 10^−2^ (middle green), 1.5 × 10^−2^ µM (dark green) on cell migration at 24 h, assessed by wound closure percentage; (**d**) Effect of CBD:PTX at 5:5 × 10^−3^ (light blue), 10:10^−2^ (middle blue), 15:1.5 × 10^−2^ µM (dark blue) on cell migration at 24 h, assessed by wound closure percentage; (**e**) Anti-migratory effect of CBD (pink), PTX (blue) and CBD:PTX (green) compared by wound closure percentage after 24 h. Scalebar: 200 µm. Statistical analysis: (**c**,**d**) one-way ANOVA followed by a post hoc Tukey’s multiple comparison test; (**e**) two-way ANOVA followed by a post hoc Tukey’s multiple comparison test. Statistical significance is shown only for the treatment type factor: * *p* < 0.05; ** *p* < 0.01; *** *p* < 0.001; **** *p* < 0.0001.

This migration assay was performed exclusively in the U-87 MG cell line, which exhibited the highest migratory capacity compared with U-373 MG cells (Figure 3). Additionally, the in ovo results confirmed consistency in antitumour efficacy for U-87 MG, with both tumour growth measurements and tumour weights showing concordant reductions following treatment. In contrast, the U-373 MG cells, which display lower invasive potential, presented an inconsistency in the in ovo analysis: while a reduction in tumour growth was observed, the tumour weight did not reflect a corresponding effect. This discrepancy underscores the rationale for performing migration assays specifically in U-87 MG cells, as this line provides a reliable and sensitive model to evaluate the anti-migratory impact of PTX and the CBD + PTX combination. Moreover, the timeframe of 24 h was selected based on the most promising results obtained in the CBD migration studies. Regarding PTX, previous studies have demonstrated its anti-migratory activity in breast, osteosarcoma and non-small lung cancer models [73,74,75]. In the present study, PTX exhibited a concentration-dependent anti-migratory effect. The 5 × 10^−3^ concentration of PTX did not produce statistically significant differences compared with the control. In contrast, the 10^−2^ and 1.5 × 10^−3^ concentrations significantly reduced cell migration, achieving wound closure values of 65.70 ± 5.48% and 45.86 ± 4.46%, respectively (Figure 9a,c). Although the anti-migratory effect of PTX was slightly lower than that of CBD at all tested concentrations, no statistically significant differences were observed between the two drugs (Figure 9e). However, the most pronounced inhibition of cell migration was obtained when CBD and PTX were administered in combination. A concentration-dependent inhibitory effect on cell migration was observed, with progressive reductions in wound closure of 59.58 ± 5.64%, 44.15 ± 8.10% and 24.54 ± 2.52% at 5:5 × 10^−3^ µM, 10:10^−2^ µM and 15:1.5 × 10^−3^ µM CBD:PTX molar ratios, respectively (Figure 9b,d). Importantly, in all cases, the combination treatment yielded a statistically significant decrease in migration compared with either monotherapy, indicating superior anti-migratory activity when both agents were co-administered (Figure 9e).

Taken together with the synergistic interactions observed in the in vitro cytotoxic assays and in ovo antitumour efficacy experiments, these findings further reinforce the therapeutic interest of the CBD + PTX combination.

### 3.5. LNCs Formulation and Characterization

Given the additive/synergistic effect of the combination of CBD and PTX observed not only in vitro but also in ovo in two distinct glioma cell lines, together with their favourable physicochemical compatibility, this combination was selected for the rational formulation design of a co-delivery nanocarrier. The antitumour effects of PTX and CBD arise from distinct, yet potentially complementary, mechanisms, which strongly support their combination. PTX primarily exerts its cytotoxic action by hyper-stabilising microtubules through binding to β-tubulin, thereby arresting cells in the G_2_/M phase of the cell cycle and inducing apoptosis via mitotic spindle dysfunction [76,77,78]. In contrast, although the precise mechanism of CBD is not yet fully elucidated, it has been shown to modulate multiple molecular targets—such as TRPV channels, VDAC1 and PPARγ—affect key survival pathways (AKT/mTOR), and induce ER stress and mitochondrial dysfunction, which together contribute to autophagy and ultimately trigger apoptosis in tumour cells [79,80].

Moreover, both drugs would benefit from encapsulation into nanocarriers, since their clinical translation is limited by poor aqueous solubility (logP CBD: 6.3; logP PTX: 3.54), necessitating the use of organic solvents for parenteral administration, which may compromise safety [81,82]. Furthermore, the therapeutic efficacy of PTX in GBM is significantly hindered by P-gp-mediated efflux, which actively restricts its penetration into the central nervous system following systemic administration and reduces its accumulation in tumour cells. This has largely precluded the integration of PTX into standard GBM treatment regimens. Collectively, these findings support the need for innovative delivery platforms that overcome their solubility issues and P-gp-mediated resistance mechanisms.

In this context, the co-encapsulation of CBD and PTX into LNCs offers a novel and effective strategy to address these limitations. First, their simultaneous exposure at the tumour site is promoted, minimizing differences in tissue distribution arising from separate administration and enhancing the overall antitumour response by enabling the manifestation of synergistic effects despite their distinct pharmacokinetic profiles. This approach can also reduce systemic toxicity by limiting off-target exposure, which is particularly relevant in antineoplastic pharmacotherapy.

Second, co-encapsulating both compounds into a single nanocarrier substantially reduces the total amount of excipients administered compared to delivering each drug in separate formulations, thereby lowering the risk of excipient-related toxicity.

Third, these nanocarriers can serve to overcome the inherent solubility issues of both drugs, since CBD and PTX are efficiently solubilized within the oily core of the LNCs, composed of Labrafac™ Lipophile WL1349, a medium-chain triglyceride.

Building on this design, whereas many previous studies have employed a dual-delivery approach using two separate oily-core LNCs to carry different drugs [83,84,85], our formulation strategy co-encapsulates both hydrophobic agents —CBD and PTX—within a single nanocarrier. This unified formulation was developed using a 7.5:1 CBD to PTX mass ratio.

Moreover, LNCs constitute a versatile platform adaptable to multiple routes of administration. In the case of GBM, two main routes of administration have been described, namely intravenous administration for systemic delivery, where the BBB must be crossed, or intracranial administration for local delivery, which mechanically bypasses the BBB. As a result, smaller nanocarriers have been deemed more suitable for systemic delivery to enhance their BBB crossing chances [86], while bigger nanocarriers have been postulated to be more suitable for local delivery to prevent BBB crossing [87].

As highlighted by Aparicio-Blanco et al. [23], LNCs prepared by the single-step PIT method, a solvent-free, low-energy and scalable process [88], enable a precise control over their particle size as a linear function of the oil/non-ionic polyethoxylated surfactant mass ratio. This single-step methodology ensures high reproducibility and operational simplicity. Kolliphor^®^ HS15 is one of those non-ionic amphiphilic surfactants described to achieve this size control [23]. To meet the requirements of the co-delivery system for either a systemic or intracranial administration for the treatment of GBM, the mass ratio between the oily phase (Labrafac^®^ Lipophile WL1349) and the surfactant (Kolliphor^®^ HS15) has been modulated. Specifically, two oil/surfactant mass ratios —0.437 (F1 for blank LNCs and F2 for co-loaded LNCs) and 1.215 (F3 for blank LNCs and F4 for co-loaded LNCs)—have been selected.

**Figure 10:**
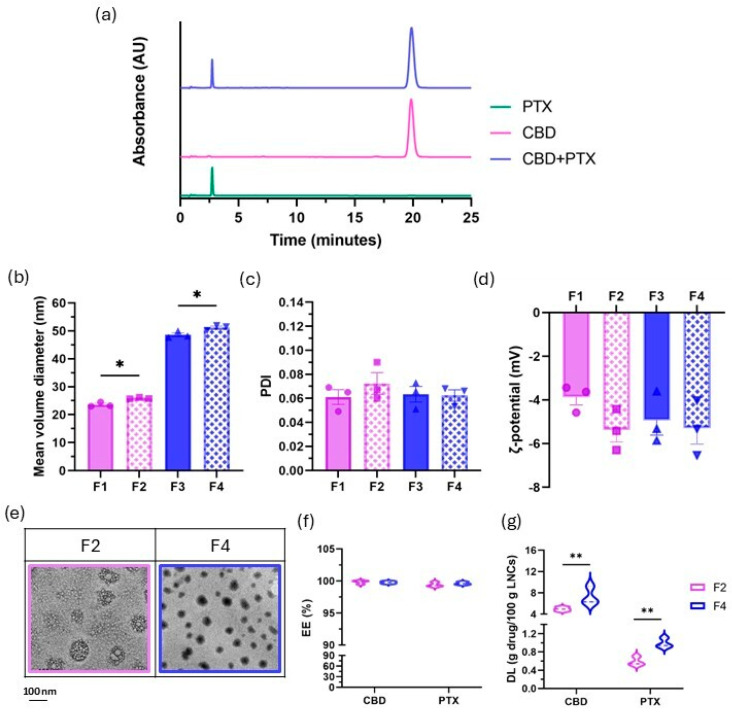
Characterization of Blank-LNCs (F1, F3) and CBD.PTX-LNCs (F2, F4). (**a**) Representative chromatogram of CBD, PTX and CBD + PTX; (**b**) Mean volume diameter (in nm) of Blank-LNCs and CBD.PTX-LNCs (n = 3); (**c**) Polydispersity index (PDI) of Blank-LNCs and CBD.PTX-LNCs (n = 3); (**d**) ζ-potential (in mV) of Blank-LNCs and CBD.PTX-LNCs (n = 3); (**e**) Representative transmission electron microscopy (TEM) images in bright field mode of F2 and F4 at 15,000×; (**f**) Encapsulation efficiency (EE, in %) of CBD and PTX in F2 and F4 (n = 3); (**g**) Drug loading (DL, in g of drug/100 g of LNCs) of CBD and PTX in F2 and F4 (n = 3). Scalebar: 100 nm. Results are expressed as mean ± SEM. Statistical analysis: (**b**,**f**,**g**) *t*-test; (**c**,**d**) Two-way ANOVA. * *p* < 0.05, ** *p* < 0.01. AU: arbitrary units.

Most analytical approaches for drug quantification from nanocarriers intended for co-delivery rely on separate protocols for each compound [89], leading to increased procedural complexity, longer analysis times, and potential lack of specificity between the two quantification methods. Interestingly, in this study, a single HPLC-UV method has been developed for the simultaneous quantification of CBD and PTX, thereby streamlining the analytical workflow and ensuring specificity. With this method, the retention time was 2.8 min for PTX and 18.9 min for CBD, in agreement with their respective logP values in reverse-phase chromatography, where greater lipophilicity results in longer retention on the stationary phase. The peaks were efficiently resolved, with a resolution coefficient of 6.2, far exceeding the 2.0 threshold for efficient chromatographic separation (Figure 10a). This method enabled the construction of simultaneous calibration curves linear over the ranges of 2–150 µg/mL for PTX (r^2^ = 0.997) and CBD (r^2^ = 0.990), which were subsequently used for quantifying encapsulation efficiency and drug loading.

Once the simultaneous analytical method had been developed, the four different formulations were prepared: blank-LNCs (F1 and F3) and CBD.PTX-LNCs (F2 and F4). Both blank and drug-loaded LNCs exhibited high inter-batch homogeneity in particle size, with coefficients of variation below 3.4%, indicating excellent reproducibility across batches (Figure 10b). Notably, co-loading with CBD and PTX led to a slight but statistically significant increase in particle size, with mean diameters of 25.9 ± 0.3 nm and 51.2 ± 0.9 nm for F2 and F4, respectively, compared to their unloaded counterparts (23.6 ± 0.8 nm and 48.6 ± 1.2 nm for F1 and F3, respectively). This increase can be attributed to the incorporation of both lipophilic drugs into the lipid core. Importantly, encapsulation did not significantly alter either the polydispersity index (PDI), with LNCs being highly monodisperse (PDI < 0.1) in all cases (Figure 10c), or surface charge, with slightly negative ζ-potential values (Figure 10d), consistent with those previously reported for drug-loaded LNCs [90,91]. Additionally, the CBD.PTX-LNCs were analysed by TEM to gain further insight into their morphology (Figure 10e). F2 appeared as cluster-like structures. This aggregation is likely related to the high surface energy and interparticle interactions of small-sized nanoparticles, which can lead to closer packing during sample drying. In contrast, F4 appeared as well-separated, individual spherical particles, as their lower surface-to-volume ratio reduces the extent of interparticle interactions and aggregation.

Next, both EE and DL were quantified with the simultaneous HPLC-UV method. Regardless of particle size, LNCs achieved EEs exceeding 99% for both PTX and CBD (Figure 10f). This remarkably high EE is likely due to the high solubility of both drugs in the medium-chain triglyceride core, which promotes effective drug incorporation within the LNCs. F2 achieved a DL of 4.96 ± 0.41 g of CBD/100 g of LNCs and 0.59 ± 0.10 g of PTX/100 g of LNCs. Meanwhile, in F4, DL was 7.30 ± 1.70 g of CBD/100 g of LNC and 0.99 ± 0.11 g of PTX/100 g of LNCs (Figure 10g). These results not only confirm the pursued 7.5:1 drug encapsulation mass ratio, but also, interestingly, reveal consistent DLs across different oil/surfactant mass ratios and hence across distinct particle sizes intended for different routes of administration. Remarkably, the LNCs developed in this study exhibited the highest DL reported to date for CBD-loaded lipid nanocarriers, in comparison with nanostructured lipid carriers [92,93,94], lipid nanoparticles [95], liposomes [96,97] and extracellular vesicles [98]. Altogether, this underscores the reproducibility and robustness of the co-loaded formulation, where particle size can be finely tuned to meet specific biopharmaceutic criteria while keeping PDI and ζ-potential unaltered. Additionally, previous studies on LNCs prepared via the same PIT method and using identical excipients have demonstrated that these LNCs maintain their particle size and high monodispersity for up to six months at both 4 °C and 25 °C as colloidal suspension [23]. In the present study, all formulations were prepared and characterized within substantially shorter time intervals, ensuring that the colloidal stability of these equivalently prepared LNCs was fully maintained throughout the experiments.

## 4. Conclusions

In this work, we rationally designed a CBD-based combination therapy through a two-step sequence aimed at developing safer and more effective cannabinoid-based strategies for GBM treatment while enhancing their translational potential. The first step focused on identifying the most synergistic and compatible CBD-chemotherapy combination for co-delivery through thorough biological and chemical screening. In the second step, two co-loaded LNC formulations were developed to enable tailored therapeutic interventions for GBM, each one intended for local or systemic administration.

A systematic in vitro screening, supported by DSC analyses, led to the rational selection of CBD and PTX as the optimal drug combination for co-delivery. This decision was based not only on their additive/synergistic antitumour effects, but also on the physicochemical compatibility required for stable co-encapsulation within LNCs. Importantly, the ability of CBD to enhance the therapeutic efficacy of PTX may allow dose reduction of the chemotherapeutic agent, potentially minimizing its associated systemic toxicity. The additive/synergistic interaction between CBD and PTX was further demonstrated in ovo and through in vitro migration assays.

The successful co-encapsulation of both lipophilic drugs into a single nanocarrier, whose particle size can be finely tuned to meet specific biopharmaceutic criteria while keeping PDI, ζ-potential and DL for both drugs unaltered, positions this LNC platform as a highly versatile delivery system for GBM treatment. The proposed CBD–PTX co-loaded LNCs offer a promising alternative platform with translational potential, supporting the development of more effective therapies for a disease still lacking curative options.

Despite the promising results presented here, future in vivo studies will be needed to evaluate each LNC size in its corresponding therapeutic context—intracranial or systemic delivery—to validate their efficacy and safety in glioma models. In vivo BBB penetration and pharmacokinetic studies will also be needed to fully understand their biodistribution.

## Figures and Tables

**Table 1:** IC_50_ values (in µM) of CBD, TMZ, BCNU, DOX and PTX in the U-87 MG and U-373 MG glioma cell lines 24 and 48 h post-treatment.

	U-87 MG		U-373 MG	
	24 h	48 h	24 h	48 h
CBD	3.7 × 10^1^ (3.4 × 10^1^–4.1 × 10^1^)	2.8 × 10^1^ (2.6 × 10^1^–3.1 × 10^1^)	2.8 × 10^1^ (2.2 × 10^1^–3.6 × 10^1^)	2.7 × 10^1^ (2.6 × 10^1^–2.9 × 10^1^)
TMZ	2.1 × 10^3^ (2 × 10^3^–2.2 × 10^3^)	1.3 × 10^3^ (8.8 × 10^2^–1.9 × 10^3^)	1.7 × 10^3^ (7.1 × 10^2^–4.1 × 10^3^)	8.2 × 10^2^ (7.5 × 10^2^–9.0 × 10^2^)
BCNU	2.1 × 10^2^ (1.6 × 10^2^–4.5 × 10^2^)	1.7 × 10^2^ (1.5 × 10^2^–2.1 × 10^2^)	3.1 × 10^2^ (2.6 × 10^2^–3.8 × 10^2^)	2.7 × 10^2^ (2.2 × 10^2^–3.4 × 10^2^)
DOX	6.1 × 10^1^ (6.1–6.1 × 10^2^)	3.5 × 10^−1^ (4.2 × 10^−2^–1.1)	6.64 (1.5–2.9 × 10^1^)	8.9 × 10^−1^ (2.2 × 10^−2^–3.5)
PTX	1.5 × 10^1^ (3.1–1.4 × 10^2^)	5.9 × 10^−2^ (1.4 × 10^−3^–9.3 × 10^−1^)	2.0 (8.5 × 10^−2^–4.9 × 10^2^)	5.2 × 10^−2^ (1.3 × 10^−2^–2 × 10^−1^)

**Table 2:** Bliss scores of CBD + TMZ, CBD + BCNU, CBD + PTX and CBD + DOX in the U-87 MG and U-373 MG glioma cell lines at 24 and 48 h post-treatment. +: no chemical incompatibility detected; –: chemically incompatible.

	Biological Screening				DSC Compatibility	Outcome
	U-87 MG		U-373 MG			
	24 h	48 h	24 h	48 h		
CBD + TMZ	3.98 ± 3.72	9.66 ± 2.46	13.76 ± 4.78	20.20 ± 3.74	–	Biologically synergistic Chemically incompatible
CBD + BCNU	−2.25 ± 5.42	−19.10 ± 5.07	−24.37 ± 4.02	−9.89 ± 4.23	–	Biologically antagonistic Chemically incompatible
CBD + DOX	−17.03 ± 6.03	−5.12 ± 3.63	−3.29 ± 5.53	−0.21 ± 5.88	–	Biologically antagonistic Chemically incompatible
CBD + PTX	11.58 ± 5.49	6.62 ± 5.49	8.08 ± 6.03	3.15 ± 3.43	+	Biologically synergistic No chemical incompatibility detected

## Data Availability

The collected and analysed datasets during this study are available from the corresponding author on reasonable request.
